# Distribution of *ABCB1* Genetic Variants and their Association with Moxifloxacin Pharmacokinetics in Hospitalized Patients with Acute Exacerbation of COPD

**DOI:** 10.2478/bjmg-2026-0011

**Published:** 2026-10-14

**Authors:** O Krstic Nakovska, M Staninova Stojovska, E Mihajloska, Godzo H, A Debreshlioska, A Tatabitovska, D Karkinski, Lj Suturkova

**Affiliations:** University Clinic of Pulmonology and Allergology, Skopje, North Macedonia; Faculty of Pharmacy, Ss. Cyril and Methodius University in Skopje, Skopje, North Macedonia

**Keywords:** *ABCB1*, acute exacerbation of chronic obstructive pulmonary disease, moxifloxacin, pharmacogenetics, pharmacokinetics, polymorphism

## Abstract

Interindividual variability in moxifloxacin pharmacokinetics may contribute to differences in therapeutic response and has been partially attributed to genetic factors affecting drug transport. The present study investigated the distribution of three common ABCB1 polymorphisms, c.1236C>T (rs1128503), c.2677G>T/A (rs2032582), and c.3435C>T (rs1045642), and explored their association with moxifloxacin pharmacokinetics in hospitalized patients with acute exacerbation of chronic obstructive pulmonary disease (AECOPD). Thirty adult patients receiving intravenous moxifloxacin 400 mg once daily were included. Plasma concentrations were quantified using a validated HPLC–UV method, and individual pharmacokinetic parameters were obtained from a previously developed population pharmacokinetic model. Genotyping was performed using TaqMan SNP Genotyping Assays. Genotype, allele and haplotype frequencies were compared with previously published data from a healthy Macedonian population. The observed genotype and allele frequencies were comparable to those reported in healthy individuals. Minor allele frequencies were 43.3% for c.1236C>T, 41.6% for c.2677G>T/A, and 45.0% for c.3435C>T. Two predominant haplotypes were identified: CGC (56.7%) and TTT (43.3%). Differences in moxifloxacin exposure were observed between genotype groups. For c.1236C>T, heterozygous carriers demonstrated the highest mean exposure, with a mean AUC of 56.24 mg·h/L and the lowest CL (9.55 L/h), whereas wild-type carriers had a lower mean AUC (37.57 mg·h/L). Similar trends were observed for c.2677G>T/A. Although limited by the small sample size, the findings suggest that *ABCB1* genetic variability may contribute to interindividual differences in moxifloxacin pharmacokinetics and warrant further investigation in larger patient cohorts.

## INTRODUCTION

Pharmacogenomics has become an important part of personalized medicine by helping to explain why patients may respond differently to the same drug. In addition to genetic factors, individualized therapeutic approaches are increasingly recognized as important in optimizing antibiotic dosing, particularly in hospitalized patients where substantial pharmacokinetic variability may occur [1]. Although pharmacogenetic-guided prescribing is increasingly used in several therapeutic areas, its application in anti-infective therapy is still limited. This is relevant because antibiotic response is often variable and may be influenced not only by the pathogen and the clinical condition of the patient, but also by host-related factors affecting drug exposure and toxicity [2].

Drug transporters are among the host factors that can influence antibiotic pharmacokinetics. By affecting absorption, tissue distribution and elimination, transporters may contribute to differences in systemic exposure between patients [3,4]. Among them, the ATP-binding cassette sub-family B member 1 (*ABCB1*), also known as multidrug resistance 1 (MDR1), is one of the most studied transporter genes. It encodes P-glycoprotein (P-gp), an ATP-dependent efflux transporter expressed in several tissues relevant for drug disposition, including the intestine, liver, kidneys and blood–brain barrier. Through its efflux activity, P-gp can limit intracellular drug accumulation and influence bioavailability, tissue penetration and elimination [3,5].

Several *ABCB1* polymorphisms have been investigated in pharmacogenetic studies. The most frequently studied variants include c.1236C>T (rs1128503), c.2677G>T/A (rs2032582), and c.3435C>T (rs1045642). These polymorphisms are common in different populations and are often inherited together as part of an *ABCB1* haplotype block [6]. Although c.1236C>T and c.3435C>T are synonymous variants, they may still affect mRNA stability, protein folding or transporter expression, while c.2677G>T/A is a non-synonymous variant that may directly influence P-gp function [7,8]. Previous studies have suggested that these variants may influence P-glycoprotein expression and activity, thereby contributing to interindividual differences in drug disposition and therapeutic response [3,7,9]. Because of these characteristics, these three polymorphisms are commonly selected when investigating the possible role of *ABCB1* in drug disposition.

Moxifloxacin is a broad-spectrum fluoroquinolone used in the treatment of respiratory tract infections, including acute exacerbations of chronic obstructive pulmonary disease (AECOPD) [10]. AECOPD is a common cause of hospitalization and antibiotic use, making optimization of antimicrobial therapy particularly important in this patient population [10,11]. Hospitalized patients frequently demonstrate substantial pharmacokinetic variability due to disease-related physiological changes, highlighting the importance of individualized antibiotic dosing strategies [12]. Moxifloxacin has favorable pharmacokinetic properties, including extensive tissue distribution and good penetration into pulmonary tissues [13,14]. However, interindividual variability in moxifloxacin exposure has been reported, including differences in maximum plasma concentration (Cmax), area under the concentration–time curve (AUC), and clearance (CL) [13,15,16]. Since moxifloxacin is a substrate of P-glycoprotein, *ABCB1* genetic variability may be one of the factors contributing to these differences. Previous studies have therefore investigated *ABCB1* polymorphisms as potential determinants of moxifloxacin pharmacokinetic variability, although available evidence remains limited and inconsistent [17].

Previous pharmacogenetic studies evaluating moxifloxacin remain limited and have reported inconsistent findings [17]. In the Republic of North Macedonia, genotype and haplotype frequencies of major *ABCB1* polymorphisms have previously been described in healthy individuals, but data in hospitalized patients with AECOPD are lacking [9]. Moreover, the potential association between *ABCB1* variants and moxifloxacin pharmacokinetics has not been evaluated in this patient population.

The aim of this study was to determine genotype and allele frequencies of *ABCB1* polymorphisms c.1236C>T, c.2677G>T/A, and c.3435C>T in hospitalized patients with AECOPD, compare them with previously published data from a healthy Macedonian population, and explore their association with individual moxifloxacin pharmacokinetic parameters, including Cmax, AUC, and CL.

## MATERIALS AND METHODS

This prospective observational study included 30 hospitalized adult patients with acute exacerbation of chronic obstructive pulmonary disease (AECOPD) admitted to the University Clinic of Pulmonology and Allergology, Skopje, North Macedonia, during 2025–2026. All patients received intravenous moxifloxacin 400 mg once daily as part of routine clinical practice. Patients with severe renal or hepatic insufficiency, known hypersensitivity to fluoroquinolones, pregnancy, lactation, recent exposure to interacting antimicrobial agents, acute cardiovascular events, active infection of another organ system, or inability to provide informed consent were excluded from the study. The study protocol was approved by the Ethics Committee of the Faculty of Pharmacy, Ss. Cyril and Methodius University in Skopje, and written informed consent was obtained from all participants prior to enrollment.

Serial blood samples were collected before infusion, immediately after completion of infusion, and at 1, 2, 3, 4, 5, 6, 7, 8, 12 and 24 h following drug administration. Plasma moxifloxacin concentrations were quantified using a validated HPLC–UV analytical method previously described by Czyrski et al. [18]. Population pharmacokinetic analysis was performed using nonlinear mixed-effects modelling implemented in MonolixSuite. Concentration–time data were best described by a two-compartment intravenous infusion model with first-order elimination. Individual pharmacokinetic parameters, including maximum plasma concentration (Cmax), area under the concentration–time curve (AUC), and clearance (CL), were derived from the final population pharmacokinetic model and subsequently used for pharmacogenetic analyses.

Peripheral blood samples were collected from all participants for pharmacogenetic analysis. Genomic DNA was isolated and analyzed at the Center for Biomolecular Pharmaceutical Analysis, Faculty of Pharmacy, Ss. Cyril and Methodius University in Skopje, North Macedonia. Genotyping of *ABCB1* polymorphisms c.1236C>T (rs1128503), c.2677G>T/A (rs2032582), and c.3435C>T (rs1045642) was performed using TaqMan SNP Genotyping Assays on an Applied Biosystems 7500 Real-Time PCR System according to the manufacturer’s instructions. Genotypes were assigned using allele-specific fluorescence detection and automated genotype calling.

Genotype and allele frequencies observed in the study population were compared with previously published data from a healthy Macedonian population reported by Naumovska et al. [9]. Pharmacokinetic parameters were evaluated according to *ABCB1* genotype groups in order to explore the potential association between genetic variability and moxifloxacin exposure.

Statistical analysis was performed using Microsoft Excel. Genotype, allele, and haplotype frequencies were calculated for each *ABCB1* polymorphism and compared descriptively with previously published data from a healthy Macedonian population. Pharmacokinetic parameters, including Cmax, AUC, and CL, were summarized according to genotype groups. Continuous variables are presented as mean ± standard deviation, whereas categorical variables are expressed as frequencies and percentages. Due to the exploratory nature of the study and the limited sample size, only descriptive analyses were performed.

## RESULTS

Thirty hospitalized patients with acute exacerbation of chronic obstructive pulmonary disease (AECOPD) were successfully genotyped for three *ABCB1* polymorphisms: c.1236C>T (rs1128503), c.2677G>T/A (rs2032582), and c.3435C>T (rs1045642).

For c.1236C>T, the wild-type genotype was observed in 11 patients (36.7%), the heterozygous genotype in 12 patients (40.0%), and the homozygous variant genotype in 7 patients (23.3%). The minor allele frequency was 43.3%, closely corresponding to the frequency previously reported in the healthy Macedonian population (43.9%) [9]. For c.2677G>T/A, the wild-type genotype was identified in 11 patients (36.7%), while 19 patients (63.3%) carried at least one variant allele (AG or AA genotype). The minor allele frequency was 41.6%, comparable to the frequency previously reported in healthy Macedonian individuals (44.3%) [9]. For c.3435C>T, the heterozygous genotype was the most frequently observed genotype, occurring in 13 patients (43.3%), followed by the wild-type genotype in 10 patients (33.3%) and the homozygous variant genotype in 7 patients (23.3%). The minor allele frequency was 45.0%, closely matching the frequency previously reported for the Macedonian population (46.0%) [9]. Genotype, allele and haplotype frequencies are presented in Table 1.

**Table 1.** Genotype, allele and haplotype frequencies of *ABCB1* polymorphisms in hospitalized patients with AECOPD

**Polymorphism**	**Genotype/Haplotype**	**n (%)**
c.1236C>T (rs1128503)	CC	11 (36.7)
	CA	12 (40.0)
	AA	7 (23.3)
	Minor allele frequency (A)	43.3
c.2677G>T/A (rs2032582)	GG	11 (36.7)
	AG/AA	19 (63.3)
	Minor allele frequency	41.6
c.3435C>T (rs1045642)	CC	10 (33.3)
	CT	13 (43.3)
	TT	7 (23.3)
	Minor allele frequency (T)	45.0
Haplotype	CGC	17 (56.7)
	TTT	13 (43.3)

Haplotype analysis identified two predominant *ABCB1* haplotypes in the study population. The wild-type CGC haplotype was observed in 17 patients (56.7%), whereas the variant TTT haplotype was present in 13 patients (43.3%). No additional haplotypes were identified in the study cohort. Overall, genotype, allele, and haplotype frequencies observed in hospitalized patients with AECOPD were comparable to those previously reported in the healthy Macedonian population [9].

The relationship between *ABCB1* genetic variability and moxifloxacin pharmacokinetic parameters was evaluated using individual estimates of maximum plasma concentration (Cmax), area under the concentration–time curve (AUC), and clearance (CL). Pharmacokinetic parameters according to genotype groups are summarized in Table 2.

**Table 2.** Moxifloxacin pharmacokinetic parameters according to **ABCB1** genotype groups

**Polymorphism**	**Genotype**	**Cmax (µg/mL), mean ± SD**	**AUC (mg·h/L), mean ± SD**	**CL (L/h), mean ± SD**
c.1236C>T (rs1128503)	CC	5.88 ± 2.16	37.57 ± 15.20	12.40 ± 5.28
	CA	6.88 ± 3.44	56.24 ± 39.68	9.55 ± 4.51
	AA	4.55 ± 1.46	43.51 ± 19.24	10.96 ± 5.01
c.2677G>T/A (rs2032582)	GG	5.85 ± 2.18	39.99 ± 13.53	11.02 ± 3.53
	AG/AA	6.63 ± 3.43	51.56 ± 40.44	11.38 ± 6.29
c.3435C>T (rs1045642)	CC	6.00 ± 2.20	48.10 ± 31.84	10.67 ± 4.54
	CT	6.57 ± 3.48	47.14 ± 31.88	10.63 ± 4.41
	TT	4.82 ± 1.35	42.72 ± 20.37	11.85 ± 6.74

Values are presented as mean ± SD. Cmax, maximum plasma concentration; AUC, area under the concentration–time curve; CL, clearance.

For c.1236C>T, heterozygous carriers demonstrated the highest moxifloxacin exposure. Patients carrying the CA genotype exhibited a mean Cmax of 6.88 μg/mL and a mean AUC of 56.24 mg·h/L, together with the lowest mean CL (9.55 L/h). In comparison, patients with the CC genotype showed a mean AUC of 37.57 mg·h/L and mean CL of 12.40 L/h, while AA carriers demonstrated intermediate values (AUC 43.51 mg·h/L; CL 10.96 L/h).

For c.2677G>T/A, carriers of variant alleles demonstrated higher moxifloxacin exposure compared with wild-type carriers. Mean AUC values were 51.56 mg·h/L in variant allele carriers and 39.99 mg·h/L in wild-type carriers. Mean CL values were 11.38 and 11.02 L/h, respectively.

For c.3435C>T, the homozygous variant genotype was associated with lower moxifloxacin exposure. Patients carrying the TT genotype exhibited lower mean AUC and Cmax values together with higher mean CL compared with CC and CT carriers. Mean AUC values were 48.10, 47.14, and 42.72 mg·h/L for CC, CT, and TT genotypes, respectively, while corresponding mean CL values were 10.67, 10.63, and 11.85 L/h.

Overall, differences in moxifloxacin exposure were observed across *ABCB1* genotype groups, with the most pronounced trend observed for c.1236C>T, where heterozygous carriers demonstrated higher AUC and lower CL values compared with both wild-type and homozygous variant carriers.

## DISCUSSION

The present study investigated the distribution of three common *ABCB1* polymorphisms (c.1236C>T (rs1128503), c.2677G>T/A (rs2032582), and c.3435C>T (rs1045642)) and their potential association with moxifloxacin pharmacokinetics in hospitalized patients with acute exacerbation of chronic obstructive pulmonary disease. Genotype, allele, and haplotype frequencies were largely comparable to those previously reported in the healthy Macedonian population [9]. In addition, differences in moxifloxacin exposure were observed among genotype groups, particularly for c.1236C>T and c.2677G>T/A, suggesting a potential contribution of *ABCB1* genetic variability to differences in moxifloxacin pharmacokinetics among patients.

The genotype and allele frequencies observed in our study closely resembled those reported by Naumovska et al. in healthy Macedonian individuals [9]. Minor allele frequencies for c.1236C>T, c.2677G>T/A, and c.3435C>T differed only marginally from published population data. Similarly, haplotype analysis demonstrated the predominance of the CGC and TTT haplotypes, which have also been described as common *ABCB1* haplotypes in different populations [6,9]. These findings indicate that the genetic background of hospitalized AECOPD patients is generally comparable to that of the broader Macedonian population.

The most pronounced pharmacokinetic differences in our study were observed for c.1236C>T, where heterozygous carriers demonstrated higher AUC values and lower clearance compared with both wild-type and homozygous variant carriers. A similar trend was observed for c.2677G>T/A, whereas c.3435C>T demonstrated a less consistent relationship with moxifloxacin exposure. Although the magnitude of these differences was modest, the observed trends suggest that *ABCB1* genetic variability may contribute to differences in systemic moxifloxacin exposure.

Our findings are consistent with the limited available evidence regarding the pharmacogenetics of moxifloxacin. Annisa et al. investigated the influence of *ABCB1* and *SLCO1B1* polymorphisms on moxifloxacin pharmacokinetics in patients with multidrug-resistant tuberculosis and similarly explored the relationship between transporter gene variability and systemic drug exposure [17]. Although direct comparison is limited by differences in study design, patient population, and sample size, both studies support the concept that genetic variability in drug transporters may contribute to interindividual variability in moxifloxacin pharmacokinetics.

The haplotype analysis further supports the observed genetic patterns. Only two major haplotypes, CGC and TTT, were identified in the study population. Previous studies have suggested that the combined effect of multiple *ABCB1* polymorphisms may be more relevant than the contribution of individual variants considered separately [6]. Although haplotype–pharmacokinetic associations were not formally evaluated in the present study, the predominance of these haplotypes is consistent with previously described inheritance patterns of *ABCB1* variants and with the strong linkage disequilibrium reported between c.1236C>T, c.2677G>T/A, and c.3435C>T [6,9].

The clinical relevance of these findings extends beyond moxifloxacin itself. As emphasized by Stocco et al., pharmacogenetic research in anti-infective therapy remains substantially less developed than in other therapeutic fields despite considerable variability in antimicrobial response [19]. While clinical factors remain the major determinants of antibiotic exposure, including pathophysiological changes associated with severe illness, genetic variability may represent an additional contributor to pharmacokinetic differences among patients [19,20].

The present study has several strengths, including the simultaneous evaluation of pharmacokinetic and pharmacogenetic data obtained from the same patient cohort and the use of pharmacokinetic parameters derived from a previously developed population pharmacokinetic model. Nevertheless, the relatively small sample size, single-center design, and evaluation of only three *ABCB1* polymorphisms limit the generalizability of the findings and preclude definitive conclusions regarding genotype–phenotype associations. The observed pharmacokinetic differences provide preliminary evidence of a potential association between *ABCB1* genetic variability and moxifloxacin exposure; however, their potential clinical implications remain to be established. Larger studies are needed to confirm these observations and further clarify the role of *ABCB1* genetic variability in moxifloxacin pharmacokinetics.

## CONCLUSION

This study demonstrated that genotype, allele, and haplotype frequencies of the *ABCB1* polymorphisms (c.1236C>T (rs1128503), c.2677G>T/A (rs2032582), and c.3435C>T (rs1045642)) in hospitalized patients with AECOPD were comparable to those previously reported in the healthy Macedonian population. Differences in moxifloxacin pharmacokinetic parameters were observed among genotype groups, particularly for c.1236C>T and c.2677G>T/A, suggesting a potential contribution of *ABCB1* genetic variability to interindividual variability in drug exposure. Although these findings should be interpreted cautiously because of the limited sample size, they add to the currently limited evidence regarding the pharmacogenetics of moxifloxacin. Further studies involving larger patient populations are needed to confirm these observations and clarify their potential clinical relevance.
